# Supplementary material for: The abilities in dog pain sign recognition as assessed by presenting seventeen listed dog behavioural signs and three case descriptions to dog owners and non-dog owners
Source: PLoS One. 2026 Apr 1;21(4):e0344512. doi: 10.1371/journal.pone.0344512 (PMC13042741; doi:10.1371/journal.pone.0344512)
Supplement: S1 File — (DOCX) [file pone.0344512.s001.docx]

**S1 File - Three cases of dogs and their behavioural changes, presented to participants**

***Case 1: Rex***

Rex, a young German Shepherd dog of six months lives in a family with children, in an apartment at the first floor. In the morning, he joins the family when the children are being brought to school. At midday he walks ten minutes and in the evening he goes for a walk with one of the adults from the family for half an hour in the nearby park. The owners followed a puppy course with Rex. At the moment he does not visit a dog school, because of the busy lives of the family members (sports of the children, work of both adults, of which one works parttime, the other fulltime). Since recently, Rex eats not thrice but twice daily. He has been dewormed, vaccinated and was recently medically checked by routine veterinary checkup. Rex likes balls and chewing on chewies.

The behaviour:

Recently, Rex has shown increased attachment behaviour. Before this, he could be home alone well. Previously he also rolled up in a snug ball in his dog basket. Now he often shadows the adult family members and lies close by them. He no longer lies rolled up in a ball. Not in his dog basket, nor anywhere else. At night Rex is restless. The family members hear him get up and lay down at another spot than he was. At times they hear him pant and scratch bedroom doors. He still wants to play with his ball, but notably shortens park walks, by turning towards the apartment sooner. Rex is eating well, likes chewing as much as before. He is not destructive, not fouling the home and no other behavioural changes were noted.

Rex is showing behavioural changes. Which motivations could underpin these changes?

- Fear
- Hormones, such as in puberty
- Learning processes
- The dog’s raising
- Pain
- Boredom

For the option above that you scored with the highest likeliness, which behavioural changes made you attribute this option?

- Increased attachment behaviour
- Shadowing adult family members
- Restlessness at night
- Not lying rolled up anymore
- Shortening the park walk

***Case 2: Coco***

Coco is a three-year-old Chihuahua living with a couple in an apartment on the eight floor, both owners are employed. The apartment is only reachable by an elevator. Coco is from a private breeder and she has been with the couple since puppyhood. She prefers wet food from a tin, which she is given daily. Every now and then she’ll eat from the kibble that is always available to her. During her annual routine veterinary checkup nothing stood out. Coco has been vaccinated and dewormed. Coco walks on a leash every morning for half an hour. Afterwards she is alone in the apartment for five hours on average. During that time, she lies predominantly on the couch. That is, this is where her owners find her upon their return. In the afternoon she goes to the park with one of her owners, where she is allowed off-leash for an hour: she likes to run and fetch balls. In the evening, she will walk on leash again for about twenty minutes.

The behaviour:

In the past weeks her owners notice that Coco is less enthusiastic to go to the park. Also, they find her on her dog cushion on the floor upon their return in the afternoon. That dog cushion was previously only used by her sporadically. During walks Coco regularly lifts her left hind leg and then hops on three legs, particularly when she runs. The first time that Coco showed this behaviour, the owners had to laugh about it. Coco looks at other dogs playing with balls, but is hardly playing with her ball herself anymore. If she does, she is also seen hopping on three legs at times.

Coco is showing behavioural changes. Which motivations could underpin these changes?

- Fear
- Hormones, such as in puberty
- Learning processes
- The dog’s raising
- Pain
- Boredom

For the option above that you scored with the highest likeliness, which behavioural changes made you attribute this option?

- Hopping
- Keeping left leg raised
- Less enthusiasm for park walk
- Lesser play with ball
- Opting for dog cushion not couch

***Case 3: Zora***

Zora is a two-year-old Jack Russell Terrier, living with a family with two children of three and six years, in a quiet neighbourhood. Zora was adopted from a shelter. She was adopted there by her current family when she was sixteen weeks old. Little to nothing is known on the first owner. Possibly, Zora was left alone a lot, after this first owner buying Zora, thus taking her from her mother and siblings. Zora had her routine veterinary checkup half a year ago and nothing stood out. Zora has been vaccinated and dewormed. Zora’s food is given in specifically weighed amounts. The owners ensure high quality food. In the morning Zora is walked extensively and in the afternoon she often plays with the family’s children in the garden. In the evening a final walk of the day is provided, of which the length depends on available time.

The behaviour:

Recently, Zora wants to go in to the garden, instead of going out for her morning walk. It does not matter whether she is accompanied into the garden or not. Whereas previously she would have a fixed routine of sniffing objects and plants, she now goes into the garden and behaves differently. She enters with a trot, stands before a wall that separates her garden from the neighbour’s garden. If her family doesn’t take her away from there, she will stand like that for at least two minutes and afterwards she will dig at the wall, as if she wants to go through the wall into the neighbour’s garden. Should anyone from the family take her indoors, then she will walk around restlessly and run towards the backyard door if someone heads towards it. However, if she falls asleep, she will sleep deeply and in the morning, when the family comes downstairs, Zora will greet them as regularly, with a broadly wagging tail, picking up toys from her toy basket and bringing these towards them.

Zora is showing behavioural changes. Which motivations could underpin these changes?

- Fear
- Hormones, such as in puberty
- Learning processes
- The dog’s raising
- Pain
- Boredom

For the option above that you scored with the highest likeliness, which behavioural changes made you attribute this option?

- Wanting to go into the garden instead of walking
- Changed sniffing routines
- Head towards/ digging at the wall
- Restlessness indoors
- Backyard door orientation
