# Supplementary material for: The abilities in dog pain sign recognition as assessed by presenting seventeen listed dog behavioural signs and three case descriptions to dog owners and non-dog owners
Source: PLoS One. 2026 Apr 1;21(4):e0344512. doi: 10.1371/journal.pone.0344512 (PMC13042741; doi:10.1371/journal.pone.0344512)
Supplement: S2 Table — (DOCX) [file pone.0344512.s002.docx]

**S2 Table - The reported previous experience with a painful accident, illness and/ or treatment in N=647 participants (N=530 dog owners, N=117 non-dog owners)**

|  | All | N=647 | Dog owners | N=530 | Non-dog owners | N=117 |
| --- | --- | --- | --- | --- | --- | --- |
| ***Accident*** |  |  |  |  |  |  |
| Yes | 25% | 160 | 27% | 32 | 24% | 128 |
| No | 72% | 469 | 72% | 84 | 73% | 385 |
| Prefer not to say | 3% | 18 | 1% | 1 | 3% | 17 |
| ***Illness*** |  |  |  |  |  |  |
| Yes | 37% | 240 | 34% | 40 | 38% | 200 |
| No | 60% | 385 | 65% | 76 | 58% | 309 |
| Prefer not to say | 3% | 22 | 1% | 1 | 4% | 21 |
| ***Treatment*** |  |  |  |  |  |  |
| Yes | 46% | 298 | 46% | 298 | 45% | 237 |
| No | 5% | 329 | 51% | 329 | 52% | 274 |
| Prefer not to say | 3% | 20 | 3% | 20 | 3% | 19 |
