# Supplementary material for: The abilities in dog pain sign recognition as assessed by presenting seventeen listed dog behavioural signs and three case descriptions to dog owners and non-dog owners
Source: PLoS One. 2026 Apr 1;21(4):e0344512. doi: 10.1371/journal.pone.0344512 (PMC13042741; doi:10.1371/journal.pone.0344512)
Supplement: S3 Table — (DOCX) [file pone.0344512.s003.docx]

**S3 Table - The reported previous experience with their dog suffering a painful accident, illness and/ or treatment in N=530 dog owners**

| Previous painful event | Percentage | N |
| --- | --- | --- |
| ***All*** |  |  |
| Yes | 55% | 290 |
| No | 45% | 240 |
| ***Accident*** |  |  |
| Yes | 88% | 469 |
| No | 12% | 61 |
| ***Illness*** |  |  |
| Yes | 72% | 382 |
| No | 28% | 148 |
| ***Treatment*** | |  |
| Yes | 67% | 354 |
| No | 33% | 176 |
