# Supplementary material for: The abilities in dog pain sign recognition as assessed by presenting seventeen listed dog behavioural signs and three case descriptions to dog owners and non-dog owners
Source: PLoS One. 2026 Apr 1;21(4):e0344512. doi: 10.1371/journal.pone.0344512 (PMC13042741; doi:10.1371/journal.pone.0344512)
Supplement: S4 Table — (DOCX) [file pone.0344512.s004.docx]

**S4 Table - The mean likeliness of a dog behavioural sign indicating pain as reported by N=647 participants (N=530 dog owners, N=117 non-dog owners), with ‘0’ indicating very unlikely and ‘4’ indicating very likely**

|  | All participants | Dog owners | Non-dog owners Mean±S.D. (range) |
| --- | --- | --- | --- |
|  | Mean±S.D. (range) | Mean±S.D. (range) |  |
| Change in personality | 3.4±0.7 (0-4) | 3.4±0.7 (0-4) | 3.3±0.7 (2-4) |
| Hesitant paw lifting | 3.4±0.9 (0-4) | 3.3±0.9 (0-4) | 3.5±0.7 (1-4) |
| Fluctuating mood | 3.3±0.8 (0-4) | 3.4±0.8 (0-4) | 3.2±0.7 (1-4) |
| Reduced play | 3.1±0.8 (0-4) | 3.1±0.8 (0-4) | 3.1±0.8 (0-4) |
| Increased scratching | 2.9±0.9 (0-4) | 3.0±0.9 (0-4) | 2.9±1.0 (0-4) |
| Coat changes | 2.9±0.9 (0-4) | 2.9±1.0 (0-4) | 2.8±1.0 (0-4) |
| Changed look | 2.7±1.0 (0-4) | 2.7±1.0 (0-4) | 2.6±0.9 (0-4) |
| Increased grooming | 2.6±1.0 (0-4) | 2.6±1.0 (0-4) | 2.5±1.1 (0-4) |
| Turn the head or body away | 2.5±1.1 (0-4) | 2.4±1.2 (0-4) | 2.8±0.9 (0-4) |
| Increased blinking | 2.4±1.1 (0-4) | 2.4±1.1 (0-4) | 2.4±1.0 (0-4) |
| Air licking | 2.3±1.1 (0-4) | 2.4±1.1 (0-4) | 2.2±1.1 (0-4) |
| Freeze | 2.3±1.7 (0-4) | 2.2±1.2 (0-4) | 2.5±1.2 (0-4) |
| Surface licking | 2.2±1.2 (0-4) | 2.3±1.2 (0-4) | 1.9±1.2 (0-4) |
| Lip licking | 2.1±1.2 (0-4) | 2.1±1.2 (0-4) | 2.1±1.1 (0-4) |
| Nose licking | 1.8±1.1 (0-4) | 1.8±1.2 (0-4) | 1.9±1.1 (0-4) |
| Yawning | 1.8±1.7 (0-4) | 1.8±1.2 (0-4) | 1.8±1.2 (0-4) |
| Air sniffing | 1.6±1.1 (0-4) | 1.5±1.1 (0-4) | 1.7±1.1 (0-4) |

**S5 Table - The likeliness percentages (N) of a dog behavioural sign indicating pain in three categories (not [very] likely, neutral, [very] likely scores) as reported by N=647 participants and comparing dog owners (N=530) to non-dog owners (N=117) with Mann-Whitney U tests**

|  | **All** |  |  | **Dog owners** | |  | **Non-dog owners** | |  |
| --- | --- | --- | --- | --- | --- | --- | --- | --- | --- |
|  | **Not (very) likely** | **Neutral** | **(Very) likely** | **Not (very) likely** | **Neutral** | **(Very) likely** | **Not (very) likely** | **Neutral** | **(Very) likely** |
| Air licking (z=-1.71, P=0.087) | 21.1% (N=137) | 31.1% (N=201) | 47.8% (N=309) | 20.19% (N=107) | 30.6% (N=162) | 49.2% (N=261) | 25.6% (N=30) | 33.3% (N=39) | 41.0% (N=48) |
| Air sniffing (z=-1.73, P=0.083) | 47.0% (N=304) | 30.8% (N=199) | 22.3% (N=144) | 48.7% (N=258) | 29.8% (N=158) | 21.5% (N=114) | 39.3% (N=46) | 35.0% (N=41) | 25.6% (N=30) |
| Change in personality (z=-0.45, P=0.656) | 2.3% (N=15) | 9.1% (N=59) | 88.6% (N=573) | 1.5% (N=8) | 8.9% (N=47) | 89.6% (N=475) | 0% (N=0) | 12.0% (N=14) | 88.0% (N=103) |
| Changed look (z=-0.30, P=0.766) | 11.1% (N=72) | 29.5% (N=191) | 59.4% (N=384) | 10.8% (N=57) | 30.4% (N=161) | 58.9% (N=312) | 12.8% (N=15) | 25.6% (N=30) | 61.5% (N=72) |
| Coat changes (z=-1.12, P=0.264) | 7.6% (N=49) | 22.0% (N=142) | 70.5% (N=456) | 7.0% (N=37) | 21.7% (N=115) | 71.3% (N=378) | 10.3% (N=12) | 23.1% (N=27) | 66.7% (n=78) |
| Fluctuating mood (z=-0.79, P=0.428) | 2.3% (N=15) | 9.1% (N=59) | 88.6% (N=573) | 2.5% (N=13) | 8.5% (N=45) | 89.1% (N=472) | 1.7% (N=2) | 12.0% (N=14) | 86.3% (N=101) |
| **Freezing (z=-2.68, P=0.007)** | 22.4% (N=145) | 31.8% (N=206) | 45.8% (N=296) | 23.4% (N=124) | 33.6% (N=178) | 43.0% (N=455) | 17.9% (N=21) | 23.9% (N=28) | 58.1% (N=68) |
| Hesitant paw lifting (z=-1.166, P=0.096) | 5.4% (N=35) | 7.7% (N=50) | 86.9% (N=562) | 6.0% (N=32) | 8.1% (N=43) | 85.8% (N=455) | 2.6% (N=3) | 6.0% (N=7) | 91.5% (N=107) |
| Increased blinking (z=-0.08, P=0.936) | 20.6% (N=133) | 28.0% (N=181) | 51.5% (N=333) | 21.7% (N=115) | 26.0% (N=138) | 52.3% (N=277) | 15.4% (N=18) | 36.8% (N=43) | 47.9% (N=56) |
| Increased grooming (z=-1.06, P=0.289) | 13.0% (N=84) | 27.4% (N=177) | 59.7% (N=386) | 12.1% (N=64) | 27.5% (N=146) | 60.4% (N=320) | 17.1% (N=20) | 26.5% (N=31) | 56.4% (N=66) |
| Increased scratching (z=-0.25, P=0.802) | 6.5% (N=42) | 18.1% (N=117) | 75.4% (N=488) | 5.8% (N=31) | 18.7% (N=99) | 75.5% (N=400) | 9.4% (N=11) | 15.4% (N=18) | 75.2% (N=88) |
| Lip licking (z=-0.49, P=0.627) | 30.5% (N=197) | 26.4% (N=171) | 43.1% (N=279) | 30.9% (N=164) | 26.2% (N=139) | 42.8% (N=227) | 28.2% (N=33) | 27.4% (N=32) | 44.4% (N=52) |
| Nose licking (z=-0.48, P=0.632) | 37.4% (N=242) | 32.8% (N=212) | 29.8% (N=193) | 37.7% (N=200) | 32.8% (N=174) | 29.4% (N=156) | 35.9% (N=42) | 32.5% (N=38) | 31.6% (N=37) |
| Reduced play (z=-0.67, P=0.502) | 3.1% (N=20) | 13.6% (N=88) | 83.3% (N=539) | 3.0% (N=16) | 13.2% (N=70) | 83.8% (N=444) | 3.4% (N=4) | 15.4% (N=18) | 81.2% (N=95) |
| **Surface licking (z=-2.68, P=0.007)** | 27.7% (N=179) | 26.1% (N=169) | 46.2% (N=299) | 25.3% (N=134) | 26.6% (N=141) | 48.1% (N=255) | 38.5% (N=45) | 23.9% (N=28) | 37.6% (N=44) |
| **Turn the head or body away (z=-3.51, P<0.001)** | 18.4% (N=119) | 27.2% (N=176) | 54.4% (N=352) | 20.9% (N=111) | 27.4% (N=145) | 51.7% (N=274) | 6.8% (N=8) | 26.5% (N=31) | 66.7% (N=78) |
| Yawning (z=-0.51, P=0.614) | 38.0% (N=246) | 29.4% (N=190) | 32.6% (N=211) | 36.6% (N=194) | 31.5% (N=167) | 31.9% (N=169) | 44.4% (N=52) | 19.7% (N=23) | 35.9% (N=42) |

**S6 Table - The likeliness percentages (N) of a dog behavioural sign indicating pain in three categories (not [very] likely, neutral, [very] likely scores) as reported by N=644 participants and comparing N=363 participants indicating to have experienced a painful event themselves with N=261 indicating not to have experienced this; with Mann-Whitney U tests**

|  | **All** |  |  | **Without painful experience** | |  | **With painful experience** | |  |
| --- | --- | --- | --- | --- | --- | --- | --- | --- | --- |
|  | **Not (very) likely** | **Neutral** | **(Very) likely** | **Not (very) likely** | **Neutral** | **(Very) likely** | **Not (very) likely** | **Neutral** | **(Very) likely** |
| Air licking (z=-0.98, P=0.328) | 21.2% (N=137) | 31.1% (N=201) | 47.8% (N=309) | 23.4% (N=61) | 29.9% (N=78) | 46.7% (N=122) | 18.7% (N=68) | 32.2% (N=117) | 49.0% (N=178) |
| Air sniffing (z=-1.27, P=0.204) | 47.0% (N=304) | 30.8% (N=199) | 22.3% (N=144) | 49.0% (N=128) | 31.0% (N=81) | 19.9% (N=52) | 44.9% (N=163) | 30.9% (N=112) | 24.2% (N=88) |
| **Change in personality (z=-2.22, P=0.027)** | 1.2% (N=8) | 9.4% (N=61) | 89.3% (N=578) | 1.5% (N=4) | 12.3% (N=32) | 86.2% (N=225) | 0.8% (N=3) | 7.4% (N=27) | 91.7% (N=333) |
| Changed look (z=-1.74, P=0.082) | 11.1% (N=72) | 29.5% (N=191) | 59.4% (N=384) | 9.6% (N=25) | 35.6% (N=93) | 54.8% (N=143) | 11.0% (N=40) | 25.6% (N=93) | 63.4% (N=230) |
| Coat changes (z=-0.20, P=0.842) | 7.6% (N=49) | 21.9% (N=142) | 70.5% (N=456) | 6.9% (N=18) | 23.4% (N=61) | 69.7% (N=182) | 7.7% (N=28) | 21.5% (N=78) | 70.8% (N=257) |
| Fluctuating mood (z=-1.36, P=0.175) | 2.3% (N=15) | 9.1% (N=59) | 88.6% (N=573) | 2.7% (N=7) | 10.7% (N=28) | 86.6% (N=226) | 1.9% (N=7) | 8.0% (N=29) | 90.1% (N=327) |
| Freezing (z=-1.47, P=0.141) | 22.4% (N=145) | 31.8% (N=206) | 45.7% (N=296) | 21.8% (N=57) | 36.0% (N=94) | 42.1% (N=110) | 21.2% (N=77) | 28.9% (N=105) | 49.9% (N=181) |
| Hesitant paw-lifting (z=-0.244, P=0.807) | 5.4% (N=35) | 7.7% (N=50) | 86.9% (N=562) | 5.0% (N=13) | 8.4% (N=22) | 86.6% (N=226) | 5.2% (N=19) | 7.4% (N=27) | 87.3% (N=317) |
| **Increased blinking (z=-2.24, P=0.025)** | 20.6% (N=133) | 28.0% (N=181) | 51.5% (N=333) | 23.0% (N=60) | 31.0% (N=81) | 46.0% (N=120) | 19.0% (N=69) | 25.3% (N=92) | 55.6% (N=202) |
| Increased grooming (z=-0.528, P=0.589) | 13.0% (N=84) | 27.4% (N=177) | 59.7% (N=386) | 13.8% (N=36) | 24.1% (N=63) | 62.1% (N=162) | 12.4% (N=45) | 28.7% (N=104) | 59.0% (N=214) |
| Increased scratching (z=-0.71, P=0.476) | 6.5% (N=42) | 18.1% (N=117) | 75.4% (N=488) | 6.9% (N=18) | 16.1% (N=42) | 77.0% (N=201) | 6.1% (N=22) | 19.8% (N=72) | 74.1% (N=269) |
| Lip licking (z=-1.61, P=0.107) | 30.4% (N=197) | 26.4% (N=171) | 43.1% (N=279) | 34.1% (N=89) | 25.3% (N=66) | 40.6% (N=106) | 27.3% (N=99) | 27.5% (N=100) | 45.2% (N=164) |
| Nose licking (z=-1.23, P=0.220) | 37.4% (N=242) | 32.8% (N=212) | 29.8% (N=193) | 39.8% (N=104) | 32.6% (N=85) | 27.6% (N=72) | 35.5% (N=129) | 33.1% (N=120) | 31.4% (N=114) |
| Reduced play (z=-1.90, P=0.057) | 3.1% (N=20) | 13.6% (N=88) | 83.3% (N=539) | 3.8% (N=10) | 16.5% (N=43) | 79.7% (N=208) | 2.2% (N=8) | 12.4% (N=45) | 85.4% (N=310) |
| Surface licking (z=-1.21, P=0.226) | 27.7% (N=179) | 26.1% (N=169) | 46.2% (N=299) | 29.1% (N=76) | 27.6% (N=72) | 43.3% (N=113) | 25.6% (N=93) | 26.4% (N=96) | 47.9% (N=174) |
| Turn the head or body away (z=-0.30, P=0.764) | 18.4% (N=119) | 27.2% (N=176) | 54.4% (N=352) | 17.2% (N=45) | 28.7% (N=75) | 54.0% (N=141) | 17.9% (N=65) | 26.2% (N=95) | 55.9% (N=203) |
| **Yawning (z=-2.05, P=0.040)** | 38.0% (N=246) | 29.4% (N=190) | 32.6% (N=211) | 41.0% (N=107) | 31.0% (N=81) | 28.0% (N=73) | 35.5% (N=129) | 27.8% (N=101) | 36.6% (N=133) |

**S7 Table - The likeliness percentages (N) of a dog behavioural sign indicating pain in three categories (not [very] likely, neutral, [very] likely scores) as reported by N=530 dog owning participants and comparing N=240 participants indicating to have a dog that previously experienced a painful event with N=290 indicating their dog not to have experienced this; with Mann-Whitney U tests**

|  | **All** |  |  | **Without painful experience** | |  | **With painful experience** | |  |
| --- | --- | --- | --- | --- | --- | --- | --- | --- | --- |
|  | **Not (very) likely** | **Neutral** | **(Very) likely** | **Not (very) likely** | **Neutral** | **(Very) likely** | **Not (very) likely** | **Neutral** | **(Very) likely** |
| Air licking (z=-1.32, P=0.185) | 20.2% (N=107) | 30.6% (N=162) | 49.2% (N=261) | 24.1% (N=70) | 27.6% (N=80) | 48.3% (N=140) | 15.4% (N=37) | 34.2% (N=82) | 50.4% (N=121) |
| Air sniffing (z=-1.00, P=0.320) | 48.7% (N=258) | 29.8% (N=158) | 21.5% (N=114) | 51.0% (N=148) | 27.9% (N=81) | 21.0% (N=61) | 45.8% (N=110) | 32.1% (N=77) | 22.1% (N=53) |
| **Change in personality (z=-1.99, P=0.046)** | 1.5% (N=8) | 8.9% (N=47) | 89.6% (N=475) | 2.1% (N=6) | 10.7% (N=31) | 87.2% (N=253) | 0.8% (N=2) | 6.7% (N=16) | 92.5% (N=222) |
| **Changed look (z=-3.46, P<0.001)** | 10.8% (N=57) | 30.4% (N=161) | 58.9% (N=312) | 12.1% (N=35) | 36.2% (N=105) | 51.7% (N=150) | 9.2% (N=22) | 23.3% (N=56) | 67.5% (N=162) |
| **Coat changes (z=-2.13, P=0.033)** | 7.0% (N=37) | 21.7% (N=115) | 71.3% (N=378) | 8.3% (N=24) | 24.1% (N=70) | 67.6% (N=196) | 5.4% (N=13) | 18.8% (N=45) | 75.8% (N=182) |
| Fluctuating mood (z=-0.92, P=0.360 | 2.5% (N=13) | 8.5% (N=45) | 89.1% (N=472) | 2.8% (N=8) | 9.3% (N=27) | 87.9% (N=255) | 2.1% (N=5) | 7.5% (N=18) | 90.4% (N=271) |
| Freezing (z=-0.38, P=0.701) | 23.4% (N=124) | 33.6% (N=178) | 43.0% (N=228) | 24.5% (N=71) | 32.8% (N=95) | 42.8% (N=124) | 22.1% (N=53) | 34.6% (N=83) | 43.3% (N=104) |
| Hesitant paw lifting (z=-1.11, P=0.267) | 6.0% (N=32) | 8.1% (N=43) | 85.8% (N=455) | 5.5% (N=16) | 10.3% (N=30) | 84.1% (N=244) | 6.7% (N=16) | 5.4% (N=13) | 87.9% (N=211) |
| **Increased blinking (z=-2.14, P=0.033)** | 21.7% (N=115) | 26.0% (N=138) | 52.3% (N=277) | 22.8% (N=66) | 30.0% (N=87) | 47.2% (N=137) | 20.4% (N=49) | 21.3% (N=51) | 58.3% (N=140) |
| **Increased grooming (z=-2.42, P=0.015)** | 12.1% (N=64) | 27.5% (N=146) | 60.4% (N=320) | 13.4% (N=39) | 31.0% (N=90) | 55.5% (N=161) | 10.4% (N=25) | 23.3% (N=56) | 66.3% (N=159) |
| **Increased scratching (z=-2.11, P=0.035)** | 5.8% (N=31) | 18.7% (N=99) | 75.5% (N=400) | 6.2% (N=18) | 22.1% (N=64) | 71.7% (N=208) | 5.4% (N=13) | 14.6% (N=35) | 80.0% (N=192) |
| Lip licking (z=-0.06, P=0.949) | 30.9% (N=164) | 26.2% (N=139) | 42.8% (N=227) | 31.0% (N=90) | 25.9% (N=75) | 43.1% (N=125) | 30.8% (N=74) | 26.7% (N=64) | 42.5% (N=102) |
| Nose licking (z=-0.03, P=0.979) | 37.7% (N=200) | 32.8% (N=174) | 29.4% (N=156) | 37.2% (N=108) | 33.8% (N=98) | 29.0% (N=84) | 38.3% (N=92) | 31.7% (N=76) | 30.0% (N=72) |
| **Reduced play (z=-4.04, P<0.001)** | 3.0% (N=16) | 13.2% (N=70) | 83.8% (N=444) | 4.5% (N=13) | 17.6% (N=51) | 77.9% (N=226) | 1.3% (N=3) | 7.9% (N=19) | 90.8% (N=218) |
| **Surface licking (z=-2.97, P=0.003)** | 25.3% (N=134) | 26.6% (N=141) | 48.1% (N=255) | 29.7% (N=86) | 27.6% (N=80) | 42.8% (N=124) | 20.0% (N=48) | 25.4% (N=61) | 54.6% (N=131) |
| Turn the head or body away (z=-1.20, P=0.232) | 20.9% (N=111) | 27.4% (N=145) | 51.7% (N=274) | 22.8% (N=66) | 27.6% (N=80) | 49.7% (N=144) | 18.8% (N=45) | 27.1% (N=65) | 54.2% (N=130) |
| Yawning (z=-0.23, P=0.820) | 36.6% (N=194) | 31.5% (N=167) | 31.9% (N=169) | 36.2% (N=105) | 33.1% (N=96) | 30.7% (N=89) | 37.1% (N=89) | 29.6% (N=71) | 33.3% (N=80) |

**S8 Table - The reported mean likeliness of a dog’s motivation for the described behaviour in three cases and the reasons for a participant selecting the motivation with the highest likeliness in N=647 participants (N=530 dog owners, N=117 non-dog owners; with ‘0’ indicating very unlikely and ‘4’ indicating very likely for the motivations and ‘0’ indicating not a reason and ‘1’ indicating a reason for the reasons for selecting a motivation at the highest likeliness)**

|  | **All (N=647)** | **Dog owners (N=530)** | **Non dog owners (N=117)** |
| --- | --- | --- | --- |
| ***Case 1: Rex*** |  |  |  |
| Fear | 2.13±1.22 (0-4) | 2.15±1.25 (0-4) | 2.04±1.10 (0-4) |
| Hormones | 1.99±1.14 (0-4) | 2.05±1.15 (0-4) | 1.72±1.05 (0-4) |
| Learning processes | 1.87±1.19 (0-4) | 1.92±1.19 (0-4) | 1.63±1.13 (0-4) |
| A dog’s raising | 1.66±1.26 (0-4) | 1.70±1.26 (0-4) | 1.49±1.28 (0-4) |
| Pain | 2.36±1.28 (0-4) | 2.35±1.29 (0-4) | 2.39±1.20 (0-4) |
| Boredom | 2.59±1.29 (0-4) | 2.62±1.30 (0-4) | 2.43±1.25 (0-4) |
| ***Possible reasons*** |  |  |  |
| Increased attachment behaviour | 0.44±0.50 (0-1) | 0.44±0.50 (0-1) | 0.44±0.50 (0-1) |
| Shadowing adult family members | 0.43±0.50 (0-1) | 0.44±0.50 (0-1) | 0.38±0.49 (0-1) |
| Restlessness at night | 0.66±0.47 (0-1) | 0.67±0.47 (0-1) | 0.64±0.48 (0-1) |
| Not lying rolled up anymore | 0.42±0.49 (0-1) | 0.43±0.50 (0-1) | 0.39±0.49 (0-1) |
| Shortening the park walk | 0.49±0.50 (0-1) | 0.49±0.50 (0-1) | 0.46±0.50 (0-1) |
| ***Case 2: Coco*** |  |  |  |
| Fear | 0.61±0.91 (0-4) | 0.61±0.90 (0-4) | 0.60±0.93 (0-4) |
| Hormones | 0.31±0.72 (0-4) | 0.29±0.69 (0-4) | 0.40±0.86 (0-4) |
| Learning processes | 0.61±0.95 (0-4) | 0.61±0.95 (0-4) | 0.62±0.94 (0-4) |
| A dog’s raising | 0.39±0.77 (0-4) | 0.37±0.76 (0-4) | 0.45±0.83 (0-3) |
| Pain | 3.71±0.71 (0-4) | 3.74±0.68 (0-4) | 3.60±0.84 (0-4) |
| Boredom | 0.56±0.98 (0-4) | 0.52±0.95 (0-4) | 0.72±1.11 (0-4) |
| ***Possible reasons*** |  |  |  |
| Hopping | 0.73±0.45 (0-1) | 0.74±0.44 (0-1) | 0.68±0.47 (0-1) |
| Keeping left leg raised | 0.89±0.32 (0-1) | 0.90±0.31 (0-1) | 0.85±0.35 (0-1) |
| Less enthusiasm for park walk | 0.66±0.47 (0-1) | 0.69±0.46 (0-1) | 0.54±0.50 (0-1) |
| Lesser play with ball | 0.67±0.47 (0-1) | 0.70±0.46 (0-1) | 0.51±0.50 (0-1) |
| Opting for dog cushion not couch | 0.69±0.46 (0-1) | 0.70±0.46 (0-1) | 0.63±0.48 (0-1) |
| ***Case 3: Zora*** |  |  |  |
| Fear | 1.15±1.33 (0-4) | 1.15±1.33 (0-4) | 1.12±1.35 (0-4) |
| Hormones | 1.46±1.45 (0-4) | 1.44±1.45 (0-4) | 1.56±1.46 (0-4) |
| Learning processes | 1.92±1.38 (0-4) | 1.94±1.39 (0-4) | 1.83±1.33 (0-4) |
| A dog’s raising | 0.86±1.13 (0-4) | 0.87±1.15 (0-4) | 0.80±1.03 (0-3) |
| Pain | 0.66±1.04 (0-4) | 0.68±1.05 (0-4) | 0.53±0.99 (0-4) |
| Boredom | 1.41±1.35 (0-4) | 1.43±1.37 (0-4) | 1.35±1.26 (0-4) |
| ***Possible reasons*** |  |  |  |
| Wanting to go into the garden instead of walking | 0.51±0.50 (0-1) | 0.53±0.50 (0-1) | 0.44±0.50 (0-1) |
| Changed sniffing routines | 0.40±0.49 (0-1) | 0.41±0.49 (0-1) | 0.36±0.48 (0-1) |
| Head/digging at the wall | 0.71±0.45 (0-1) | 0.73±0.44 (0-1) | 0.62±0.49 (0-1) |
| Restlessness indoors | 0.62±0.49 (0-1) | 0.63±0.48 (0-1) | 0.56±0.50 (0-1) |
| Backyard door orientation | 0.52±0.50 (0-1) | 0.54±0.50 (0-1) | 0.46±0.50 (0-1) |

**S9 Table – The reasons provided for attributing pain or learning processes as a motivation for described dog behaviour in three cases**

We presented participants with three cases. Case 1 described a dog with more subtle pain signs (based on panosteitis). Case 2 described a dog with less subtle pain signs, more directly related to movement ability (based on patella luxation). Case 3 described a dog with learning processes related behaviour (based on prey drive after neighbours got two rabbits). We asked participants to indicate how likely they felt described behaviours per case were indicative of motivation of pain, boredom, fear, hormones (e.g. puberty), learning processes (conditioning), or the dog’s raising. Next, we asked the participants to indicate for the choice they gave the highest likeliness rating which behaviours made them opt for it. We indicate with Mann-Whitney U tests the differences in selecting a behaviour (yes=1) or not selecting it (no=0), for selecting the pain motivation for each of the cases and for selecting the learning processes motivation for case 3 (P<0.05). Participants could select more than one behaviour as a reason for selecting a motivation.

***Case 1 – Dog with more subtle pain signs***

| **Behaviour indicative to motivation** | **Pain deemed causal (N=347)** |  | **Pain not deemed causal (N=300)** |  | **Mann-Whitney U test (z, P-value)** |
| --- | --- | --- | --- | --- | --- |
|  | Reason indicated (yes) | Reason not indicated (no) | Reason indicated (yes) | Reason not indicated (no) |  |
| Increased attachment behaviour | 35% (N=120) | 65% (N=227) | 45% (N=136) | 55% (N=164) | z=-5.13, P<0.001 |
| Shadowing adult family members | 32% (N=110) | 68% (N=237) | 56% (N=167) | 44% (N=133) | z=-6.14, P<0.001 |
| Restlessness at night | 67% (N=234) | 33% (N=113) | 65% (N=196) | 35% (N=104) | z=-0.56, P=0.573 |
| Not lying rolled up anymore | 62% (N=215) | 38% (N=132) | 19% (N=58) | 81% (N=242) | z=-10.94, P<0.001 |
| Shortening the park walk | 65% (N=227) | 35% (N=120) | 29% (N=87) | 71% (N=213) | z=-9.13, P<0.001 |

***Case 2 – Dog with less subtle pain signs, more directly related to movement ability***

| **Behaviour indicative to motivation** | **Pain deemed causal (N=620)** |  | **Pain not deemed causal (N=27)** |  | **Mann-Whitney U test (z, P-value)** |
| --- | --- | --- | --- | --- | --- |
|  | Reason indicated (yes) | Reason not indic. (no) | Reason indicated (yes) | Reason not indic. (no) |  |
| Hopping | 74% (N=461) | 26% (N=159) | 37% (N=10) | 63% (N=17) | z=-4.26, P<0.001 |
| Keeping left leg raised | 90% (N=555) | 10% (N=65) | 26% (N=7) | 74% (N=20) | z=-2.50, P=0.013 |
| Less enthusiasm for park walk | 67% (N=418) | 33% (N=202) | 41% (N=11) | 59% (N=16) | z=-2.87, P=0.004 |
| Lesser play with ball | 68% (N=423) | 32% (N=197) | 37% (N=10) | 63% (N=17) | z=-3.37, P<0.001 |
| Opting for dog cushion not couch | 70% (N=437) | 30% (N=183) | 33% (N=9) | 67% (N=18) | z=-4.08, P<0.001 |

***Case 3 – Dog with learning processes related behaviour***

| **Behaviour indicative to motivation** | **Pain deemed causal (N=55)** |  | **Pain not deemed causal (N=592)** |  | **Mann-Whitney U test (z, P-value)** |
| --- | --- | --- | --- | --- | --- |
|  | Reason indicated (yes) | Reason not indic. (no) | Reason indicated (yes) | Reason not indic. (no) |  |
| Wanting to go into the garden | 55% (N=30) | 45% (N=25) | 51% (N=301) | 49% (N=291) | z=-0.53, P=0.600 |
| Changed sniffing routines | 38% (N=21) | 62% (N=34) | 40% (N=237) | 60% (N=355) | z=-0.27, P=0.789 |
| Head/digging at the wall | 65% (N=36) | 35% (N=19) | 72% (N=425) | 28% (N=167) | z=-0.99, P=0.321 |
| Restlessness indoors | 76% (N=42) | 24% (N=13) | 60% (N=356) | 40% (N=236) | z=-3.36, P=0.018 |
| Backyard door orientation | 35% (N=19) | 65% (N=36) | 54% (N=319) | 46% (N=273) | z=-2.75, P=0.006 |

***Case 3 – Dog with learning processes related behaviour***

| **Behaviour indicative to motivation** | **Learning deemed causal (N=271)** |  | **Learning not deemed causal (N=376)** |  | **Mann-Whitney U test (z, P-value)** |
| --- | --- | --- | --- | --- | --- |
|  | Reason indicated (yes) | Reason not indic. (no) | Reason indicated (yes) | Reason not indic. (no) |  |
| Wanting to go into the garden | 59% (N=160) | 41% (N=111) | 45% (N=171) | 55% (N=205) | z=-3.40, P<0.001 |
| Changed sniffing routines | 42% (N=113) | 58% (N=158) | 39% (N=145) | 61% (N=231) | z=-0.80, P=0.422 |
| Head/digging at the wall | 77% (N=210) | 23% (N=61) | 67% (N=251) | 33% (N=125) | z=-2.97, P=0.003 |
| Restlessness indoors | 60% (N=162) | 40% (N=108) | 63% (N=235) | 38% (N=141) | z=-0.61, P=0.554 |
| Backyard door orientation | 62% (N=168) | 38% (N=103) | 45% (N=170) | 55% (N=206) | z=-4.21, P<0.001 |
