# Supplementary material for: The abilities in dog pain sign recognition as assessed by presenting seventeen listed dog behavioural signs and three case descriptions to dog owners and non-dog owners
Source: PLoS One. 2026 Apr 1;21(4):e0344512. doi: 10.1371/journal.pone.0344512 (PMC13042741; doi:10.1371/journal.pone.0344512)
Supplement: S5 Table — (DOCX) [file pone.0344512.s005.docx]

**S5 Table - The likeliness percentages (N) of a dog behavioural sign indicating pain in three categories (not [very] likely, neutral, [very] likely scores) as reported by N=647 participants and comparing dog owners (N=530) to non-dog owners (N=117) with Mann-Whitney U tests**

|  | **All** |  |  | **Dog owners** | |  | **Non-dog owners** | |  |
| --- | --- | --- | --- | --- | --- | --- | --- | --- | --- |
|  | **Not (very) likely** | **Neutral** | **(Very) likely** | **Not (very) likely** | **Neutral** | **(Very) likely** | **Not (very) likely** | **Neutral** | **(Very) likely** |
| Air licking (z=-1.71, P=0.087) | 21.1% (N=137) | 31.1% (N=201) | 47.8% (N=309) | 20.19% (N=107) | 30.6% (N=162) | 49.2% (N=261) | 25.6% (N=30) | 33.3% (N=39) | 41.0% (N=48) |
| Air sniffing (z=-1.73, P=0.083) | 47.0% (N=304) | 30.8% (N=199) | 22.3% (N=144) | 48.7% (N=258) | 29.8% (N=158) | 21.5% (N=114) | 39.3% (N=46) | 35.0% (N=41) | 25.6% (N=30) |
| Change in personality (z=-0.45, P=0.656) | 2.3% (N=15) | 9.1% (N=59) | 88.6% (N=573) | 1.5% (N=8) | 8.9% (N=47) | 89.6% (N=475) | 0% (N=0) | 12.0% (N=14) | 88.0% (N=103) |
| Changed look (z=-0.30, P=0.766) | 11.1% (N=72) | 29.5% (N=191) | 59.4% (N=384) | 10.8% (N=57) | 30.4% (N=161) | 58.9% (N=312) | 12.8% (N=15) | 25.6% (N=30) | 61.5% (N=72) |
| Coat changes (z=-1.12, P=0.264) | 7.6% (N=49) | 22.0% (N=142) | 70.5% (N=456) | 7.0% (N=37) | 21.7% (N=115) | 71.3% (N=378) | 10.3% (N=12) | 23.1% (N=27) | 66.7% (n=78) |
| Fluctuating mood (z=-0.79, P=0.428) | 2.3% (N=15) | 9.1% (N=59) | 88.6% (N=573) | 2.5% (N=13) | 8.5% (N=45) | 89.1% (N=472) | 1.7% (N=2) | 12.0% (N=14) | 86.3% (N=101) |
| **Freezing (z=-2.68, P=0.007)** | 22.4% (N=145) | 31.8% (N=206) | 45.8% (N=296) | 23.4% (N=124) | 33.6% (N=178) | 43.0% (N=455) | 17.9% (N=21) | 23.9% (N=28) | 58.1% (N=68) |
| Hesitant paw lifting (z=-1.166, P=0.096) | 5.4% (N=35) | 7.7% (N=50) | 86.9% (N=562) | 6.0% (N=32) | 8.1% (N=43) | 85.8% (N=455) | 2.6% (N=3) | 6.0% (N=7) | 91.5% (N=107) |
| Increased blinking (z=-0.08, P=0.936) | 20.6% (N=133) | 28.0% (N=181) | 51.5% (N=333) | 21.7% (N=115) | 26.0% (N=138) | 52.3% (N=277) | 15.4% (N=18) | 36.8% (N=43) | 47.9% (N=56) |
| Increased grooming (z=-1.06, P=0.289) | 13.0% (N=84) | 27.4% (N=177) | 59.7% (N=386) | 12.1% (N=64) | 27.5% (N=146) | 60.4% (N=320) | 17.1% (N=20) | 26.5% (N=31) | 56.4% (N=66) |
| Increased scratching (z=-0.25, P=0.802) | 6.5% (N=42) | 18.1% (N=117) | 75.4% (N=488) | 5.8% (N=31) | 18.7% (N=99) | 75.5% (N=400) | 9.4% (N=11) | 15.4% (N=18) | 75.2% (N=88) |
| Lip licking (z=-0.49, P=0.627) | 30.5% (N=197) | 26.4% (N=171) | 43.1% (N=279) | 30.9% (N=164) | 26.2% (N=139) | 42.8% (N=227) | 28.2% (N=33) | 27.4% (N=32) | 44.4% (N=52) |
| Nose licking (z=-0.48, P=0.632) | 37.4% (N=242) | 32.8% (N=212) | 29.8% (N=193) | 37.7% (N=200) | 32.8% (N=174) | 29.4% (N=156) | 35.9% (N=42) | 32.5% (N=38) | 31.6% (N=37) |
| Reduced play (z=-0.67, P=0.502) | 3.1% (N=20) | 13.6% (N=88) | 83.3% (N=539) | 3.0% (N=16) | 13.2% (N=70) | 83.8% (N=444) | 3.4% (N=4) | 15.4% (N=18) | 81.2% (N=95) |
| **Surface licking (z=-2.68, P=0.007)** | 27.7% (N=179) | 26.1% (N=169) | 46.2% (N=299) | 25.3% (N=134) | 26.6% (N=141) | 48.1% (N=255) | 38.5% (N=45) | 23.9% (N=28) | 37.6% (N=44) |
| **Turn the head or body away (z=-3.51, P<0.001)** | 18.4% (N=119) | 27.2% (N=176) | 54.4% (N=352) | 20.9% (N=111) | 27.4% (N=145) | 51.7% (N=274) | 6.8% (N=8) | 26.5% (N=31) | 66.7% (N=78) |
| Yawning (z=-0.51, P=0.614) | 38.0% (N=246) | 29.4% (N=190) | 32.6% (N=211) | 36.6% (N=194) | 31.5% (N=167) | 31.9% (N=169) | 44.4% (N=52) | 19.7% (N=23) | 35.9% (N=42) |
