# Supplementary material for: The abilities in dog pain sign recognition as assessed by presenting seventeen listed dog behavioural signs and three case descriptions to dog owners and non-dog owners
Source: PLoS One. 2026 Apr 1;21(4):e0344512. doi: 10.1371/journal.pone.0344512 (PMC13042741; doi:10.1371/journal.pone.0344512)
Supplement: S6 Table — (DOCX) [file pone.0344512.s006.docx]

**S6 Table - The likeliness percentages (N) of a dog behavioural sign indicating pain in three categories (not [very] likely, neutral, [very] likely scores) as reported by N=644 participants and comparing N=363 participants indicating to have experienced a painful event themselves with N=261 indicating not to have experienced this; with Mann-Whitney U tests**

|  | **All** |  |  | **Without painful experience** | |  | **With painful experience** | |  |
| --- | --- | --- | --- | --- | --- | --- | --- | --- | --- |
|  | **Not (very) likely** | **Neutral** | **(Very) likely** | **Not (very) likely** | **Neutral** | **(Very) likely** | **Not (very) likely** | **Neutral** | **(Very) likely** |
| Air licking (z=-0.98, P=0.328) | 21.2% (N=137) | 31.1% (N=201) | 47.8% (N=309) | 23.4% (N=61) | 29.9% (N=78) | 46.7% (N=122) | 18.7% (N=68) | 32.2% (N=117) | 49.0% (N=178) |
| Air sniffing (z=-1.27, P=0.204) | 47.0% (N=304) | 30.8% (N=199) | 22.3% (N=144) | 49.0% (N=128) | 31.0% (N=81) | 19.9% (N=52) | 44.9% (N=163) | 30.9% (N=112) | 24.2% (N=88) |
| **Change in personality (z=-2.22, P=0.027)** | 1.2% (N=8) | 9.4% (N=61) | 89.3% (N=578) | 1.5% (N=4) | 12.3% (N=32) | 86.2% (N=225) | 0.8% (N=3) | 7.4% (N=27) | 91.7% (N=333) |
| Changed look (z=-1.74, P=0.082) | 11.1% (N=72) | 29.5% (N=191) | 59.4% (N=384) | 9.6% (N=25) | 35.6% (N=93) | 54.8% (N=143) | 11.0% (N=40) | 25.6% (N=93) | 63.4% (N=230) |
| Coat changes (z=-0.20, P=0.842) | 7.6% (N=49) | 21.9% (N=142) | 70.5% (N=456) | 6.9% (N=18) | 23.4% (N=61) | 69.7% (N=182) | 7.7% (N=28) | 21.5% (N=78) | 70.8% (N=257) |
| Fluctuating mood (z=-1.36, P=0.175) | 2.3% (N=15) | 9.1% (N=59) | 88.6% (N=573) | 2.7% (N=7) | 10.7% (N=28) | 86.6% (N=226) | 1.9% (N=7) | 8.0% (N=29) | 90.1% (N=327) |
| Freezing (z=-1.47, P=0.141) | 22.4% (N=145) | 31.8% (N=206) | 45.7% (N=296) | 21.8% (N=57) | 36.0% (N=94) | 42.1% (N=110) | 21.2% (N=77) | 28.9% (N=105) | 49.9% (N=181) |
| Hesitant paw-lifting (z=-0.244, P=0.807) | 5.4% (N=35) | 7.7% (N=50) | 86.9% (N=562) | 5.0% (N=13) | 8.4% (N=22) | 86.6% (N=226) | 5.2% (N=19) | 7.4% (N=27) | 87.3% (N=317) |
| **Increased blinking (z=-2.24, P=0.025)** | 20.6% (N=133) | 28.0% (N=181) | 51.5% (N=333) | 23.0% (N=60) | 31.0% (N=81) | 46.0% (N=120) | 19.0% (N=69) | 25.3% (N=92) | 55.6% (N=202) |
| Increased grooming (z=-0.528, P=0.589) | 13.0% (N=84) | 27.4% (N=177) | 59.7% (N=386) | 13.8% (N=36) | 24.1% (N=63) | 62.1% (N=162) | 12.4% (N=45) | 28.7% (N=104) | 59.0% (N=214) |
| Increased scratching (z=-0.71, P=0.476) | 6.5% (N=42) | 18.1% (N=117) | 75.4% (N=488) | 6.9% (N=18) | 16.1% (N=42) | 77.0% (N=201) | 6.1% (N=22) | 19.8% (N=72) | 74.1% (N=269) |
| Lip licking (z=-1.61, P=0.107) | 30.4% (N=197) | 26.4% (N=171) | 43.1% (N=279) | 34.1% (N=89) | 25.3% (N=66) | 40.6% (N=106) | 27.3% (N=99) | 27.5% (N=100) | 45.2% (N=164) |
| Nose licking (z=-1.23, P=0.220) | 37.4% (N=242) | 32.8% (N=212) | 29.8% (N=193) | 39.8% (N=104) | 32.6% (N=85) | 27.6% (N=72) | 35.5% (N=129) | 33.1% (N=120) | 31.4% (N=114) |
| Reduced play (z=-1.90, P=0.057) | 3.1% (N=20) | 13.6% (N=88) | 83.3% (N=539) | 3.8% (N=10) | 16.5% (N=43) | 79.7% (N=208) | 2.2% (N=8) | 12.4% (N=45) | 85.4% (N=310) |
| Surface licking (z=-1.21, P=0.226) | 27.7% (N=179) | 26.1% (N=169) | 46.2% (N=299) | 29.1% (N=76) | 27.6% (N=72) | 43.3% (N=113) | 25.6% (N=93) | 26.4% (N=96) | 47.9% (N=174) |
| Turn the head or body away (z=-0.30, P=0.764) | 18.4% (N=119) | 27.2% (N=176) | 54.4% (N=352) | 17.2% (N=45) | 28.7% (N=75) | 54.0% (N=141) | 17.9% (N=65) | 26.2% (N=95) | 55.9% (N=203) |
| **Yawning (z=-2.05, P=0.040)** | 38.0% (N=246) | 29.4% (N=190) | 32.6% (N=211) | 41.0% (N=107) | 31.0% (N=81) | 28.0% (N=73) | 35.5% (N=129) | 27.8% (N=101) | 36.6% (N=133) |
