# Supplementary material for: The abilities in dog pain sign recognition as assessed by presenting seventeen listed dog behavioural signs and three case descriptions to dog owners and non-dog owners
Source: PLoS One. 2026 Apr 1;21(4):e0344512. doi: 10.1371/journal.pone.0344512 (PMC13042741; doi:10.1371/journal.pone.0344512)
Supplement: S7 Table — (DOCX) [file pone.0344512.s007.docx]

**S7 Table - The likeliness percentages (N) of a dog behavioural sign indicating pain in three categories (not [very] likely, neutral, [very] likely scores) as reported by N=530 dog owning participants and comparing N=240 participants indicating to have a dog that previously experienced a painful event with N=290 indicating their dog not to have experienced this; with Mann-Whitney U tests**

|  | **All** |  |  | **Without painful experience** | |  | **With painful experience** | |  |
| --- | --- | --- | --- | --- | --- | --- | --- | --- | --- |
|  | **Not (very) likely** | **Neutral** | **(Very) likely** | **Not (very) likely** | **Neutral** | **(Very) likely** | **Not (very) likely** | **Neutral** | **(Very) likely** |
| Air licking (z=-1.32, P=0.185) | 20.2% (N=107) | 30.6% (N=162) | 49.2% (N=261) | 24.1% (N=70) | 27.6% (N=80) | 48.3% (N=140) | 15.4% (N=37) | 34.2% (N=82) | 50.4% (N=121) |
| Air sniffing (z=-1.00, P=0.320) | 48.7% (N=258) | 29.8% (N=158) | 21.5% (N=114) | 51.0% (N=148) | 27.9% (N=81) | 21.0% (N=61) | 45.8% (N=110) | 32.1% (N=77) | 22.1% (N=53) |
| **Change in personality (z=-1.99, P=0.046)** | 1.5% (N=8) | 8.9% (N=47) | 89.6% (N=475) | 2.1% (N=6) | 10.7% (N=31) | 87.2% (N=253) | 0.8% (N=2) | 6.7% (N=16) | 92.5% (N=222) |
| **Changed look (z=-3.46, P<0.001)** | 10.8% (N=57) | 30.4% (N=161) | 58.9% (N=312) | 12.1% (N=35) | 36.2% (N=105) | 51.7% (N=150) | 9.2% (N=22) | 23.3% (N=56) | 67.5% (N=162) |
| **Coat changes (z=-2.13, P=0.033)** | 7.0% (N=37) | 21.7% (N=115) | 71.3% (N=378) | 8.3% (N=24) | 24.1% (N=70) | 67.6% (N=196) | 5.4% (N=13) | 18.8% (N=45) | 75.8% (N=182) |
| Fluctuating mood (z=-0.92, P=0.360 | 2.5% (N=13) | 8.5% (N=45) | 89.1% (N=472) | 2.8% (N=8) | 9.3% (N=27) | 87.9% (N=255) | 2.1% (N=5) | 7.5% (N=18) | 90.4% (N=271) |
| Freezing (z=-0.38, P=0.701) | 23.4% (N=124) | 33.6% (N=178) | 43.0% (N=228) | 24.5% (N=71) | 32.8% (N=95) | 42.8% (N=124) | 22.1% (N=53) | 34.6% (N=83) | 43.3% (N=104) |
| Hesitant paw lifting (z=-1.11, P=0.267) | 6.0% (N=32) | 8.1% (N=43) | 85.8% (N=455) | 5.5% (N=16) | 10.3% (N=30) | 84.1% (N=244) | 6.7% (N=16) | 5.4% (N=13) | 87.9% (N=211) |
| **Increased blinking (z=-2.14, P=0.033)** | 21.7% (N=115) | 26.0% (N=138) | 52.3% (N=277) | 22.8% (N=66) | 30.0% (N=87) | 47.2% (N=137) | 20.4% (N=49) | 21.3% (N=51) | 58.3% (N=140) |
| **Increased grooming (z=-2.42, P=0.015)** | 12.1% (N=64) | 27.5% (N=146) | 60.4% (N=320) | 13.4% (N=39) | 31.0% (N=90) | 55.5% (N=161) | 10.4% (N=25) | 23.3% (N=56) | 66.3% (N=159) |
| **Increased scratching (z=-2.11, P=0.035)** | 5.8% (N=31) | 18.7% (N=99) | 75.5% (N=400) | 6.2% (N=18) | 22.1% (N=64) | 71.7% (N=208) | 5.4% (N=13) | 14.6% (N=35) | 80.0% (N=192) |
| Lip licking (z=-0.06, P=0.949) | 30.9% (N=164) | 26.2% (N=139) | 42.8% (N=227) | 31.0% (N=90) | 25.9% (N=75) | 43.1% (N=125) | 30.8% (N=74) | 26.7% (N=64) | 42.5% (N=102) |
| Nose licking (z=-0.03, P=0.979) | 37.7% (N=200) | 32.8% (N=174) | 29.4% (N=156) | 37.2% (N=108) | 33.8% (N=98) | 29.0% (N=84) | 38.3% (N=92) | 31.7% (N=76) | 30.0% (N=72) |
| **Reduced play (z=-4.04, P<0.001)** | 3.0% (N=16) | 13.2% (N=70) | 83.8% (N=444) | 4.5% (N=13) | 17.6% (N=51) | 77.9% (N=226) | 1.3% (N=3) | 7.9% (N=19) | 90.8% (N=218) |
| **Surface licking (z=-2.97, P=0.003)** | 25.3% (N=134) | 26.6% (N=141) | 48.1% (N=255) | 29.7% (N=86) | 27.6% (N=80) | 42.8% (N=124) | 20.0% (N=48) | 25.4% (N=61) | 54.6% (N=131) |
| Turn the head or body away (z=-1.20, P=0.232) | 20.9% (N=111) | 27.4% (N=145) | 51.7% (N=274) | 22.8% (N=66) | 27.6% (N=80) | 49.7% (N=144) | 18.8% (N=45) | 27.1% (N=65) | 54.2% (N=130) |
| Yawning (z=-0.23, P=0.820) | 36.6% (N=194) | 31.5% (N=167) | 31.9% (N=169) | 36.2% (N=105) | 33.1% (N=96) | 30.7% (N=89) | 37.1% (N=89) | 29.6% (N=71) | 33.3% (N=80) |
