# Supplementary material for: The abilities in dog pain sign recognition as assessed by presenting seventeen listed dog behavioural signs and three case descriptions to dog owners and non-dog owners
Source: PLoS One. 2026 Apr 1;21(4):e0344512. doi: 10.1371/journal.pone.0344512 (PMC13042741; doi:10.1371/journal.pone.0344512)
Supplement: S8 Table — (DOCX) [file pone.0344512.s008.docx]

**S8 Table - The reported mean likeliness of a dog’s motivation for the described behaviour in three cases and the reasons for a participant selecting the motivation with the highest likeliness in N=647 participants (N=530 dog owners, N=117 non-dog owners; with ‘0’ indicating very unlikely and ‘4’ indicating very likely for the motivations and ‘0’ indicating not a reason and ‘1’ indicating a reason for the reasons for selecting a motivation at the highest likeliness)**

|  | **All (N=647)** | **Dog owners (N=530)** | **Non dog owners (N=117)** |
| --- | --- | --- | --- |
| ***Case 1: Rex*** |  |  |  |
| Fear | 2.13±1.22 (0-4) | 2.15±1.25 (0-4) | 2.04±1.10 (0-4) |
| Hormones | 1.99±1.14 (0-4) | 2.05±1.15 (0-4) | 1.72±1.05 (0-4) |
| Learning processes | 1.87±1.19 (0-4) | 1.92±1.19 (0-4) | 1.63±1.13 (0-4) |
| A dog’s raising | 1.66±1.26 (0-4) | 1.70±1.26 (0-4) | 1.49±1.28 (0-4) |
| Pain | 2.36±1.28 (0-4) | 2.35±1.29 (0-4) | 2.39±1.20 (0-4) |
| Boredom | 2.59±1.29 (0-4) | 2.62±1.30 (0-4) | 2.43±1.25 (0-4) |
| ***Possible reasons*** |  |  |  |
| Increased attachment behaviour | 0.44±0.50 (0-1) | 0.44±0.50 (0-1) | 0.44±0.50 (0-1) |
| Shadowing adult family members | 0.43±0.50 (0-1) | 0.44±0.50 (0-1) | 0.38±0.49 (0-1) |
| Restlessness at night | 0.66±0.47 (0-1) | 0.67±0.47 (0-1) | 0.64±0.48 (0-1) |
| Not lying rolled up anymore | 0.42±0.49 (0-1) | 0.43±0.50 (0-1) | 0.39±0.49 (0-1) |
| Shortening the park walk | 0.49±0.50 (0-1) | 0.49±0.50 (0-1) | 0.46±0.50 (0-1) |
| ***Case 2: Coco*** |  |  |  |
| Fear | 0.61±0.91 (0-4) | 0.61±0.90 (0-4) | 0.60±0.93 (0-4) |
| Hormones | 0.31±0.72 (0-4) | 0.29±0.69 (0-4) | 0.40±0.86 (0-4) |
| Learning processes | 0.61±0.95 (0-4) | 0.61±0.95 (0-4) | 0.62±0.94 (0-4) |
| A dog’s raising | 0.39±0.77 (0-4) | 0.37±0.76 (0-4) | 0.45±0.83 (0-3) |
| Pain | 3.71±0.71 (0-4) | 3.74±0.68 (0-4) | 3.60±0.84 (0-4) |
| Boredom | 0.56±0.98 (0-4) | 0.52±0.95 (0-4) | 0.72±1.11 (0-4) |
| ***Possible reasons*** |  |  |  |
| Hopping | 0.73±0.45 (0-1) | 0.74±0.44 (0-1) | 0.68±0.47 (0-1) |
| Keeping left leg raised | 0.89±0.32 (0-1) | 0.90±0.31 (0-1) | 0.85±0.35 (0-1) |
| Less enthusiasm for park walk | 0.66±0.47 (0-1) | 0.69±0.46 (0-1) | 0.54±0.50 (0-1) |
| Lesser play with ball | 0.67±0.47 (0-1) | 0.70±0.46 (0-1) | 0.51±0.50 (0-1) |
| Opting for dog cushion not couch | 0.69±0.46 (0-1) | 0.70±0.46 (0-1) | 0.63±0.48 (0-1) |
| ***Case 3: Zora*** |  |  |  |
| Fear | 1.15±1.33 (0-4) | 1.15±1.33 (0-4) | 1.12±1.35 (0-4) |
| Hormones | 1.46±1.45 (0-4) | 1.44±1.45 (0-4) | 1.56±1.46 (0-4) |
| Learning processes | 1.92±1.38 (0-4) | 1.94±1.39 (0-4) | 1.83±1.33 (0-4) |
| A dog’s raising | 0.86±1.13 (0-4) | 0.87±1.15 (0-4) | 0.80±1.03 (0-3) |
| Pain | 0.66±1.04 (0-4) | 0.68±1.05 (0-4) | 0.53±0.99 (0-4) |
| Boredom | 1.41±1.35 (0-4) | 1.43±1.37 (0-4) | 1.35±1.26 (0-4) |
| ***Possible reasons*** |  |  |  |
| Wanting to go into the garden instead of walking | 0.51±0.50 (0-1) | 0.53±0.50 (0-1) | 0.44±0.50 (0-1) |
| Changed sniffing routines | 0.40±0.49 (0-1) | 0.41±0.49 (0-1) | 0.36±0.48 (0-1) |
| Head/digging at the wall | 0.71±0.45 (0-1) | 0.73±0.44 (0-1) | 0.62±0.49 (0-1) |
| Restlessness indoors | 0.62±0.49 (0-1) | 0.63±0.48 (0-1) | 0.56±0.50 (0-1) |
| Backyard door orientation | 0.52±0.50 (0-1) | 0.54±0.50 (0-1) | 0.46±0.50 (0-1) |
