# Supplementary material for: The abilities in dog pain sign recognition as assessed by presenting seventeen listed dog behavioural signs and three case descriptions to dog owners and non-dog owners
Source: PLoS One. 2026 Apr 1;21(4):e0344512. doi: 10.1371/journal.pone.0344512 (PMC13042741; doi:10.1371/journal.pone.0344512)
Supplement: S9 Table — (DOCX) [file pone.0344512.s009.docx]

**S9 Table – The reasons provided for attributing pain or learning processes as a motivation for described dog behaviour in three cases**

We presented participants with three cases. Case 1 described a dog with more subtle pain signs (based on panosteitis). Case 2 described a dog with less subtle pain signs, more directly related to movement ability (based on patella luxation). Case 3 described a dog with learning processes related behaviour (based on prey drive after neighbours got two rabbits). We asked participants to indicate how likely they felt described behaviours per case were indicative of motivation of pain, boredom, fear, hormones (e.g. puberty), learning processes (conditioning), or the dog’s raising. Next, we asked the participants to indicate for the choice they gave the highest likeliness rating which behaviours made them opt for it. We indicate with Mann-Whitney U tests the differences in selecting a behaviour (yes=1) or not selecting it (no=0), for selecting the pain motivation for each of the cases and for selecting the learning processes motivation for case 3 (P<0.05). Participants could select more than one behaviour as a reason for selecting a motivation.

***Case 1 – Dog with more subtle pain signs***

| **Behaviour indicative to motivation** | **Pain deemed causal (N=347)** |  | **Pain not deemed causal (N=300)** |  | **Mann-Whitney U test (z, P-value)** |
| --- | --- | --- | --- | --- | --- |
|  | Reason indicated (yes) | Reason not indicated (no) | Reason indicated (yes) | Reason not indicated (no) |  |
| Increased attachment behaviour | 35% (N=120) | 65% (N=227) | 45% (N=136) | 55% (N=164) | z=-5.13, P<0.001 |
| Shadowing adult family members | 32% (N=110) | 68% (N=237) | 56% (N=167) | 44% (N=133) | z=-6.14, P<0.001 |
| Restlessness at night | 67% (N=234) | 33% (N=113) | 65% (N=196) | 35% (N=104) | z=-0.56, P=0.573 |
| Not lying rolled up anymore | 62% (N=215) | 38% (N=132) | 19% (N=58) | 81% (N=242) | z=-10.94, P<0.001 |
| Shortening the park walk | 65% (N=227) | 35% (N=120) | 29% (N=87) | 71% (N=213) | z=-9.13, P<0.001 |

***Case 2 – Dog with less subtle pain signs, more directly related to movement ability***

| **Behaviour indicative to motivation** | **Pain deemed causal (N=620)** |  | **Pain not deemed causal (N=27)** |  | **Mann-Whitney U test (z, P-value)** |
| --- | --- | --- | --- | --- | --- |
|  | Reason indicated (yes) | Reason not indic. (no) | Reason indicated (yes) | Reason not indic. (no) |  |
| Hopping | 74% (N=461) | 26% (N=159) | 37% (N=10) | 63% (N=17) | z=-4.26, P<0.001 |
| Keeping left leg raised | 90% (N=555) | 10% (N=65) | 26% (N=7) | 74% (N=20) | z=-2.50, P=0.013 |
| Less enthusiasm for park walk | 67% (N=418) | 33% (N=202) | 41% (N=11) | 59% (N=16) | z=-2.87, P=0.004 |
| Lesser play with ball | 68% (N=423) | 32% (N=197) | 37% (N=10) | 63% (N=17) | z=-3.37, P<0.001 |
| Opting for dog cushion not couch | 70% (N=437) | 30% (N=183) | 33% (N=9) | 67% (N=18) | z=-4.08, P<0.001 |

***Case 3 – Dog with learning processes related behaviour - PAIN***

| **Behaviour indicative to motivation** | **Pain deemed causal (N=55)** |  | **Pain not deemed causal (N=592)** |  | **Mann-Whitney U test (z, P-value)** |
| --- | --- | --- | --- | --- | --- |
|  | Reason indicated (yes) | Reason not indic. (no) | Reason indicated (yes) | Reason not indic. (no) |  |
| Wanting to go into the garden | 55% (N=30) | 45% (N=25) | 51% (N=301) | 49% (N=291) | z=-0.53, P=0.600 |
| Changed sniffing routines | 38% (N=21) | 62% (N=34) | 40% (N=237) | 60% (N=355) | z=-0.27, P=0.789 |
| Head/digging at the wall | 65% (N=36) | 35% (N=19) | 72% (N=425) | 28% (N=167) | z=-0.99, P=0.321 |
| Restlessness indoors | 76% (N=42) | 24% (N=13) | 60% (N=356) | 40% (N=236) | z=-3.36, P=0.018 |
| Backyard door orientation | 35% (N=19) | 65% (N=36) | 54% (N=319) | 46% (N=273) | z=-2.75, P=0.006 |

***Case 3 – Dog with learning processes related behaviour - LEARNING***

| **Behaviour indicative to motivation** | **Learning deemed causal (N=271)** |  | **Learning not deemed causal (N=376)** |  | **Mann-Whitney U test (z, P-value)** |
| --- | --- | --- | --- | --- | --- |
|  | Reason indicated (yes) | Reason not indic. (no) | Reason indicated (yes) | Reason not indic. (no) |  |
| Wanting to go into the garden | 59% (N=160) | 41% (N=111) | 45% (N=171) | 55% (N=205) | z=-3.40, P<0.001 |
| Changed sniffing routines | 42% (N=113) | 58% (N=158) | 39% (N=145) | 61% (N=231) | z=-0.80, P=0.422 |
| Head/digging at the wall | 77% (N=210) | 23% (N=61) | 67% (N=251) | 33% (N=125) | z=-2.97, P=0.003 |
| Restlessness indoors | 60% (N=162) | 40% (N=108) | 63% (N=235) | 38% (N=141) | z=-0.61, P=0.554 |
| Backyard door orientation | 62% (N=168) | 38% (N=103) | 45% (N=170) | 55% (N=206) | z=-4.21, P<0.001 |
