# Supplementary material for: The abilities in dog pain sign recognition as assessed by presenting seventeen listed dog behavioural signs and three case descriptions to dog owners and non-dog owners
Source: PLoS One. 2026 Apr 1;21(4):e0344512. doi: 10.1371/journal.pone.0344512 (PMC13042741; doi:10.1371/journal.pone.0344512)
Supplement: Attachment — Submitted filename: PlosOne_Response_0503.docx [file pone.0344512.s010.docx]

Amsterdam, March 5^th^, 2025

Dear Editors,

Thank you for your inquiry regarding the ethical approval of our study. We have filled in the ‘Ethics statement’ and provide all information regarding ethical approval of our study and participant consent in the methods section of the main manuscript on page 7, line 150 – 164.

Our study ‘Dog pain sign recognition in dog owners and non-dog owners’ was part of a larger project investigating dog’s acute pain sign recognition, that was reviewed by the Science-Geosciences Ethics Review Board of Utrecht University and approved on 22nd of May, 2024, the approval number being: ERB Review Vet S-24.002.

Participants were invited to take part, through an advertisement that was distributed via several social media platforms using a link providing access to the online questionnaire. Participants were informed of the anonymous collection of the data and asked to tick a box to confirm consent if they wished to partake or closed the browser if they did not which to partake.

We look forward to your decision on our manuscript and are available for answering any further questions.

Kind regards,

Silvia Gardeweg
