# Supplementary material for: The abilities in dog pain sign recognition as assessed by presenting seventeen listed dog behavioural signs and three case descriptions to dog owners and non-dog owners
Source: PLoS One. 2026 Apr 1;21(4):e0344512. doi: 10.1371/journal.pone.0344512 (PMC13042741; doi:10.1371/journal.pone.0344512)
Supplement: Attachment — Submitted filename: PLOS ONE Rebuttal 2025-09-06.docx [file pone.0344512.s011.docx]

Utrecht, September 6th, 2025

Dear Mrs. Olsson,

Thank you again to yourself and the reviewers, for assessing the manuscript ‘The abilities in dog pain sign recognition as assessed by presenting seventeen listed dog behavioural signs and three case descriptions to dog owners and non-dog owners’ for publication in PLOS ONE.

We have made the specific adaptations requested by Reviewer #1 and have further edited sentences that could be convoluted and challenging to understand.

We look forward to the publication.

Dr. Silvia Gardeweg, Dionne Picard and Ineke van Herwijnen
