# Supplementary material for: The abilities in dog pain sign recognition as assessed by presenting seventeen listed dog behavioural signs and three case descriptions to dog owners and non-dog owners
Source: PLoS One. 2026 Apr 1;21(4):e0344512. doi: 10.1371/journal.pone.0344512 (PMC13042741; doi:10.1371/journal.pone.0344512)
Supplement: Attachment — Submitted filename: Response to reviewers.docx [file pone.0344512.s012.docx]

Utrecht, November 18^th^, 2025

Dear Academic Editor and Reviewers,

Thank you for your response that allows us to improve our manuscript titled ‘The abilities in dog pain sign recognition as assessed by presenting seventeen listed dog behavioural signs and three case descriptions to dog owners and non-dog owners’. We have carefully considered the provided feedback. Please find here below, our response to each point raised. Please note that line indications refer to the manuscript without track changes, for your easy reading.

Sincerely,

Ineke R. van Herwijnen Ph.D.

i.r.vanherwijnen@uu.nl

Division of Animals in Science and Society, Faculty of Veterinary Medicine, Department of Population Health Sciences, Utrecht University, Utrecht, The Netherlands

***Response to each point raised:***

*Reviewer #1: The study is very interesting and highlights the importance of studying differences of experiences with dogs and pain when assessing knowledge of dog behaviour. The results are presented very clearly and there is a sound discussion of limitations and future implications of the findings. I would suggest a few minor changes to improve readability in some areas and clarify some points which could be mis-understood. Please see my comments below:*
**Thank you for your time, the kind feedback and suggestions for improvements.**

*51 – sentence starting “If interactions in risk contexts…” needs to be re-written as it is difficult to understand. Possibly* *Adequate recognition of warning signs from dog body language may prevent or stop risky interactions from occurring.*
**Thank you for this suggestion which we used to replace the sentence.**

*56 – I would change “science” has provided insights to "studies" or "research" or “in recent years there have been new insights”…*
**Thank you, we have replaced ‘science has’ for ‘studies have’**

*67 – re-word the first sentence of the paragraph to clarify. Example, The lack of studies exploring the ability of dog an non-dog owners in interpreting …*
**Thank you for this suggestion which we used to replace the first part of the sentence.**

*70 – It is great that there is a mention that dog bites may be caused by pain. Could you expand and add a citation? E.g.* *When in pain dogs may behave unpredictably and react to stimuli that they usually would not react to. Mills, D.S., Demontigny-Bédard, I., Gruen, M., Klinck, M.P., McPeake, K.J., Barcelos, A.M., Hewison, L., Van Haevermaet, H., Denenberg, S., Hauser, H. and Koch, C., 2020. Pain and problem behavior in cats and dogs. Animals, 10(2), p.318.*
**Thank you for the suggestion, which has been added (with the reference).**

*75 – It is unclear what “more subtle” is referring to. More subtle pain sign than what? Maybe remove more?*
**Thank you, we have removed ‘more’ in ‘more subtle’ throughout.**

*161: the sentences describing how participants were recruited is very informative, but I think it could be written in a clearer way. The word “indicating” could be replaced with “…**describing that the study was about dog behaviour without specifying the study aim to assess pain recognition”.*
**Thank you for this suggestion which we used to replace the last part of the sentence.**

*164 – For readability maybe use partake only once in the sentence.*
**Indeed and we replaced the second ‘to partake’ by ‘to do so’.**

*198- "that is" change to "aged between"*
**Thank you, ‘that is’ was removed.**

*365 – comma not needed*
**Thank you, comma removed.**

*398 - comma not needed*
**Thank you, comma removed.**

*400 – reword for clarity – e.g. “ in dogs the licking of surfaces is less…than turning of the head/body or freezing”*
**Thank you for this suggestion which we used to replace the first part of the sentence.**

*402 – remove “a dog’s” & 408 - remove “a dog’s”*
**Removed.**

*409 – recognised more than what? Maybe write as being most recognised by….?*
**Thank you for pointing this out, the sentence was adapted to ‘stood out for their relatively high levels of recognition’.**

*496 – the final sentence of the discussion is a bit difficult to follow as it is long. Is the main point to suggest dog owners receive education focusing not only on fear/stress recognition but also on recognising subtle signs of pain? Maybe then reiterate in a separate sentence the last point that this education may result in potentially benefitting….*

**Thank you for addressing this. The last sentences now read: ‘**We suggest that dog owner education also addresses dog pain recognition, with a particular focus on subtle pain signs. In doing so, dog owners may be provided with early warning signs of pain. Early recognition of pain in dogs will benefit dog welfare and may also prevent behavioural problems such as aggression.’

**Reviewer #2:**

*Reviewer #2: Thank you for the extensive revisions. The manuscript is much improved, but I would like to follow up on several key comments which I would like to further discuss.*
**Thank you for your time and extensive feedback. We have addressed this as following.**

*- L170: Why was “neutral” lumped with “very unlikely” and “unlikely” when it is the middle point between those options and “likely”/”very likely”?
Author response: Thank you for indicating this omission in the methods section. We have explained this in the methods now, line 184 onwards.
Reviewer response: While I understand that the author argues that neutral would count as “not indicating pain”, that may not be how a participant interpreted this category, since it is “neutral” and not one of the “unlikely” categories. A participant may have selected “neutral” where a behaviour could be indicative of pain in certain circumstances, but of fear (for example) in other circumstances and therefore chose the “Neutral” category. Given that the authors do not collapse the ordinal scale into a binary variable in other parts of the paper (means and standard deviations are reported), it seems odd that they would choose to do so here instead of using the full information they have available from their ordinal scale.
- L170-174: Why not use a Mann – Whitney U test so you don’t need to collapse the “neutral” category in with the “Unlikely” category? By using a chi square test you’re losing granularity in the data and also treating the data as nominal rather than ordinal.
Author response: We opted for the binary testing as this is in line with our study objective and we have many elements of testing, risking a too detailed text. To benefit reading and understanding, in line with our hypotheses and research question, this choice was made. We have now more clearly indicated this in the methods section line 184 onwards.
Reviewer response: Could the author please clarify what is meant by “we have many elements of testing, risking a too detailed text?” It’s not clear how a Mann-Whitney U test is harder to explain than a chi-square test and given that it’s appropriate for ordinal data, it would remove the need for explaining how/why ordinal data was collapsed into a binary variable. This would likely make the text shorter. A Mann-Whitney U test would tell you if there is a difference in how likely a member from group A is to consider a behaviour to be a sign of pain, compared to someone from group B. It’s not clear to me how this does not correctly align with the study objective? What you’ve said here also goes against the way you use mean and SD in other parts of the manuscript (e.g., lines 232-234 “Using the list of seventeen possible pain signs as explained in the Methods section, we assessed to which extent these were seen likely as indicative of pain by calculating means +/- S.D.”)*
**We now present data split in three categories and using Mann-Whitney U tests.**

*- L196-197: How did you account for this difference in age distributions between non-dog owners and owners in your analysis comparing scoring between dog-owners and non-dog owners? I.e., how do you know differences in the groups are due to ownership status and not age?
Author response: Thank you for pointing this out. As our study’s aim was not to indicate owner age differences in pain recognition, we feel that adding another section to the already complex results incorporating a section where age splits are made and tested, will not be in line with study aim and further complicate our results section. This is why we opted to clearly point out in the discussion this skewness. In line with your valid comment, we have added to the discussion section (line 464 onwards) the suggestion for future research that looks into age and other possible participant characteristics that could factor in, such as dog school attendance as to ensure this limitation of our study is given sufficient attention by the readers of the article (and hoping the suggestion will be picked up in future studies of course!)
Reviewer response: I understand that there are already a lot of comparisons being run and splitting by age would be adding to this number.* *However, in lines 374 – 406 the results of the dog owner versus non-dog owner analyses are discussed, but the issue of the substantial differences in age distribution are not addressed at all. This is potentially misleading for a reader who is only skimming the paper and has skipped to the discussion, as this difference in demographics between dog owners and non-dog owners is a major limitation. The purpose of my comment was not that we should necessarily look into age effects (as this is not the variable of interest here), but that it is an issue that the results are discussed in a way that doesn't highlight the other major difference between the two groups. In this large section on the first hypothesis, the difference in age distributions between the two groups should be clearly mentioned and it should be pointed out that as this analysis cannot determine causal effect and that, difference between the group of dog owners versus non dog owners could be related other factors such as age. While this is mentioned briefly in the limitations (lines 449-454) I would prefer to see this discussed as part of the discussion section on that specific hypothesis, as this is a specific limitation of that analysis and significantly impacts interpretability. If someone wanted to address this in future, it would need to be done using something like a causal inference approach.*
**Thank you for this valuable comment and additional explanation of what could be improved. In an effort to keep the structure of the Discussion section, with limitations all in one section and keeping the discussion of the limitation on our skewed age distribution there, we have in addition to stressing the skewed age (and gender) distribution in the result section now also highlighted this in the summary on outcomes with which we start the Discussion section as following (lines 403-408):**

**‘We found proof confirming our first hypothesis that dog owners do not recognise subtle dog pain signs more so than non-dog owners. However, we stress that our sample of dog owners and non-dog owners differed in demographic characteristics, such as age, as discussed below when we provide detail on the limitations of this study. Taking into account these demographic differences, dog owners achieved better results for identifying (…)’**

*Additional reviewer comments:
The author should clarify how they accounted for multiple comparisons. They mention a correction for multiple comparisons in the text, but it is not clear if it is applied correctly. Using the data they provided, I ran a chi-square using the author's methods (grouping neutral with unlikely) for the dog owner vs non-dog owner comparison. The proportion of owners/non-owners identifying the signs as potentially indicative of pain are the same as what they report, but the chi-square statistics and p-values I’m getting are different with no correction applied. This would indicate to me that a correction was applied, but it’s particularly confusing that they report a p-value for both freezing and turning head/body away that is larger than what I’ve found (this could be because they accounted for multiple comparisons), but the p-value they report for licking surfaces is smaller than the p-value I calculated. So, if they’re correcting for multiple comparisons, it’s not clear how this is done, since whatever they’re adjusting their p-values by should be the same for all rows of the table (i.e., all behaviours assessed)? P-values I calculated (with no correction for multiple comparisons for the three behaviours in question):
PS_freez: p = 0.00417; PS_turnaw: p = 0.00452; PS_licksurf = 0.04992. P-values reported in the paper (according to methods section, corrected for multiple comparisons): Freezing: p = 0.03; Turning head or body away: p = 0.03; Licking surfaces: p = 0.04. The authors reported in lines 187-189 that Bonferroni method adjusted p-values were used. However, from my understanding in that case the p-values should all have been adjusted using the same multiplier, which it appears they have not been. Could the authors please clarify how this correction was applied?*

**As we now present results with statistical testing via Mann-Whitney U tests, for which SPSS® does not include an option for Bonferonni correction, we have corrected manually, providing detail on the approach the Methods section lines 191-197.**
